# Quality Evaluation of Pseudostellariae Radix Based on Simultaneous Determination of Multiple Bioactive Components Combined with Grey Relational Analysis

**DOI:** 10.3390/molecules22010013

**Published:** 2016-12-26

**Authors:** Yujiao Hua, Shengnan Wang, Chuan Chai, Zixiu Liu, Xunhong Liu, Lisi Zou, Qinan Wu, Hui Zhao, Yan Ying

**Affiliations:** College of Pharmacy, Nanjing University of Chinese Medicine, Nanjing 210023, China; 20141550@njucm.edu.cn (Y.H.); jshmwsn@163.com (S.W.); echo_0523@hotmail.com (C.C.); liuzixiu3221@126.com (Z.L.); zlstcm@126.com (L.Z.); qnwyjs@163.com (Q.W.); zhaohui_199301@163.com (H.Z.); yanying93ly@163.com (Y.Y.)

**Keywords:** Pseudostellariae Radix, UFLC-QTRAP-MS/MS, multiple bioactive components, simultaneous quantitation, GRA

## Abstract

Pseudostellariae Radix (PR) is an important traditional Chinese herbal medicine (TCM) with vast clinical consumption because of its positive effects. However, little attention has been devoted to simultaneous analysis of its bioactive components for quality control of PR based on its different harvesting times, different growing habitats, and different processing methods. In this research, the quality of PR was evaluated based on simultaneous determination of multiple bioactive components combined with grey relational analysis (GRA). A reliable method based on ultra-fast liquid chromatography tandem triple quadrupole mass spectrometry (UFLC-QTRAP-MS/MS) was established to simultaneously determine the contents of 30 components in PR, including two cyclopeptides, 12 nucleosides, and 16 amino acids. Furthermore, grey relational analysis was performed to evaluate the quality of PR samples according to the contents of these 30 components. The results showed that the quality of PR harvested in 6 August 2013, cultivated in Jurong, Jiangsu, and treated by oven drying 60 °C was better than that of other PR samples. The proposed method is useful for the overall assessment on the quality of PR, and this study provides valuable information for revealing the dynamic change laws of metabolite accumulation in PR and choosing the most suitable harvesting time and reasonable processing method of PR to obtain the best quality.

## 1. Introduction

Pseudostellariae Radix (PR) is the dry tuberous root of *Pseudostellaria heterophylla* (Miq.) Pax ex Pax et Hoffm. [1]. It is a staple traditional Chinese herbal medicine which has the functions of strengthening the spleen, replenishing Qi, moistening lungs, and producing fluids. This medicinal herb is widely consumed clinically for its positive effects. It is reported that PR can be used for inappetence [2], thirst [3], debility [4], diabetes [5], and weakness after illness [6], and it has become an important medicine to cure loss of appetite in children due to spleen deficiency. Phytochemical studies indicate that cyclopeptides, amino acids, and nucleosides are the main bioactive compounds in PR [7,8,9]. Some studies have proved that these PR components have various biological activities. For example, cyclopeptides inhibit melaninogenesis and tyrosinase activity [10]; amino acids are the important medical components in PR, which have nourishing and tonifying functions [11]; nucleosides are the active ingredients that enhance immune activity [12]. The synergistic action of these components is considered to be responsible for the broad clinical effects of PR. As PR resources are declining and the demand for original medicinal materials is rising annually, the government has established a large-scale cultivation base for PR. However, due to differences in the ecological environment, the accumulation of active components and the quality of the PR material from different habitats show great differences. The harvesting time and primary processing of PR are the important factors which are closely related to the quality of this TCM. The different harvesting times and various processing methods can affect the chemical components in PR. On account of different harvesting times, different habitats, and different processing methods, it is difficult to achieve their standardization to ensure the effectiveness in clinical use. In view of the current situation, it is necessary to develop a rapid and reliable method to evaluate the bioactive components for quality control of PR.

In recent years, a variety of analytical methods have focused on the determination of the contents of cyclopeptides or nucleosides or amino acids in PR. High-performance liquid chromatography (HPLC) [13], gas chromatography coupled to mass spectrometry (GC/MS) [14], and high performance capillary electrophoresis (HPCE) [15] have all been reported as tools for PR quality assessment. However, the contents of only cyclopeptides or nucleosides or amino acids cannot accurately represent the quality of the complex herbal products, and a method for the simultaneous quantitative determination of cyclopeptides, nucleosides, and amino acids has not been established. Therefore, a universal method for the quantitative determination of multiple components in PR is necessary and convenient for their quality control. The ultra-fast liquid chromatography tandem triple quadrupole mass spectrometry (UFLC-QTRAP-MS/MS) method, which uses multiple reaction monitoring (MRM) for quantitative analysis, is highly sensitive and fast. MRM is a data acquisition technology with high sensitivity, accuracy, and throughput, which uses one transition each from the precursor to the reporter ions for every compound, respectively. This technology has been widely used in the analysis of complex TCMs [16,17,18].

In this work, an accurate and reliable method based on UFLC-QTRAP-MS/MS for the simultaneous determination of multiple bioactive components in PR was established and validated. A total of 30 compounds, including two cyclopeptides, 12 nucleosides, and 16 amino acids were selected as the marker compounds. Multiple reaction monitoring (MRM), a tandem MS scan mode unique to triple quadrupole MS instrumentation, was employed for quantification in the present study. The proposed method was successfully applied to analyze fifteen PR samples from different harvesting times, different habitats, and treated with different processing methods. Furthermore, grey relational analysis (GRA), a quantitative comparative analysis method which is wildly used in the quality assessment in TCMs [19,20], was performed to evaluate the samples according to the contents of the 30 marker compounds. This method does not need too much sample size and typical distribution, and also has the advantage of needing a small amount of computation, and the results will accord with the quantitative results. To the best of our knowledge, this is the most comprehensive published report in the quantitative analysis of PR.

## 2. Results and Discussion

### 2.1. Optimization of Extraction Conditions

The extraction conditions, including extraction method (ultrasonic extraction and refluxing extraction), extraction solvent (water, 25% methanol, 50% methanol, 75% methanol, and 100% methanol), solvent to sample ratios (80:1, 100:1, 200:1, and 250:1 (*v*/*w*)), and extraction time (45 min, 60 min, 75 min, and 90 min) were optimized in order to obtain the most satisfactory extraction efficiency. The results showed that ultrasonic extraction with a 200:1 ratio of water for 60 min at room temperature was sufficient for complete extraction of the target compounds.

### 2.2. Optimization of UFLC Conditions

The amino acids, nucleosides, and cyclopeptides in PR all have large hydrophilicity, so hydrophilic chromatography columns have a strong retention ability as well as good resolution for these compounds; thus, in order to achieve rapid and efficient analysis, an XBridge Amide (2.1 mm × 100 mm, 3.5 μm) column (Waters, Milford, MA, USA) was employed for this analysis. Different mobile phases (including acetonitrile–water, methanol–water, acetonitrile–formic acid solution, methanol–formic acid solution, acetonitrile with formic acid solution–formic acid solution, and methanol with formic acid solution–formic acid solution), flow rate (0.4, 0.5, and 0.6 mL/min) as well as column temperature (25, 30, and 35 °C) were examined and compared. As a result, it was determined that acetonitrile with 0.2% formic acid–0.2% formic acid solution at a flow rate of 0.6 mL/min with the column temperature of 30 °C resulted in satisfactory separation in a short analysis time.

### 2.3. Optimization of MS Conditions

In order to develop a sensitive and accurate quantitative method, all the compounds were examined separately in direct infusion mode by a full-scan MS method in both positive and negative mode, and all analytes showed maximum sensitivity when the instrument was operated in the positive ion mode. The parameters of fragmentor voltage (FV) and collision energy (CE) were optimized to achieve the most abundant, specific, and stable transition for each compound. The retention time (RT) and MS information for each analytes including [M + H]^+^, precursor and product ions, FV and CE are shown in Table 1.

### 2.4. UFLC Method Validation

All method validations for quantification were performed using the UFLC-QTRAP-MS/MS technique. The data of each method validation are summarized in Table 2. The calibration curves exhibited good linearity (*r*^2^ > 0.9947) within the test range.

The LODs and LOQs ranged between 0.13 ng/mL and 31.13 ng/mL and 0.40 ng/mL to 93.40 ng/mL, respectively. The intra- and inter-day precision RSD values ranged from 0.61% to 3.34% and 1.93% to 4.39%, respectively. The satisfactory repeatability presented as RSDs were in the range from 1.01% to 3.93%. The solution stability presented as RSD was less than 3.99%, indicating the sample was stable when stored at room temperature for 24 h. The recoveries varied between 95.68% and 104.85%, with RSDs less than 4.83%, demonstrating that this method was validated for all kinds of analytes.

### 2.5. Quantification of Cyclopeptides, Nucleosides, and Amino Acids

**Figure 1:**
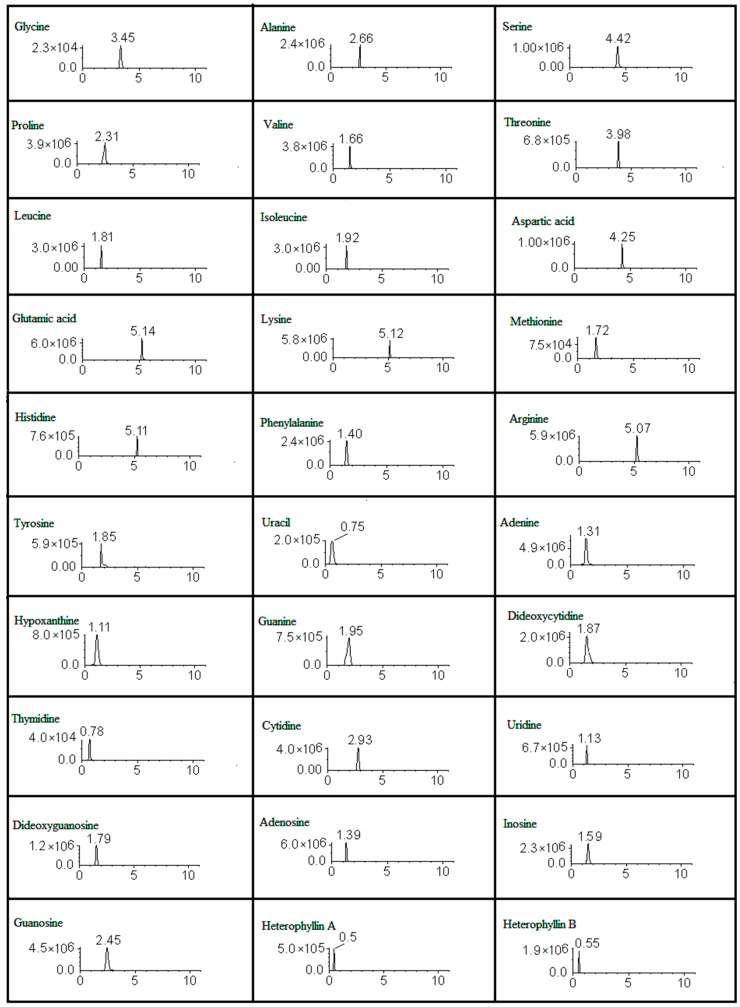
Representative extract ions chromatograms (XIC) of multiple-reaction monitoring (MRM) chromatograms of the 30 investigated compounds.

The validated analytical method was successfully applied to the simultaneous determination of two cyclopeptides, 16 amino acids, and 12 nucleosides in PR preparations containing 15 samples from different harvesting times, different habitats, and dealt with different processing methods. Each sample was determined three times and the results were reported as mean ± SD. The total contents of each type of compounds (including cyclopeptides, amino acids, and nucleosides) were statistically evaluated by one-way ANOVA analysis. Typical MRM chromatograms are shown in Figure 1 and the quantitative results are presented in Table 3.

By comparing the amounts, it was found that the compounds of PR from different harvesting times, different habitats, and dealt with using different processing methods were quite different. For different harvesting times, it was clearly shown that the total contents of 30 compounds varied from 3951.04 μg/g to 7858.10 μg/g, and in the following order: (highest) 2013.8.6 > 2013.6.15 > 2013.9.12 > 2013.7.9 > 2013.7.15 (lowest).The total contents of each type of compounds were also calculated, the levels of the two cyclopeptides, with total contents of 414.47 μg/g, 16 amino acids with total contents of 6158.95 μg/g, and 12 nucleosides with total contents of 1284.68 μg/g in PR harvested on 6 August 2013 were significantly higher than that in the herbal materials from other harvesting times based on the significant difference test. As for the habitats, the total contents of 30 constituents ranged from 4267.30 μg/g to 6580.65 μg/g, and in order: (highest) Jurong, Jiangsu > Zherong 2, Fujian > Xuancheng, Anhui > Shibing, Guizhou > Zhengrong 1, Fujian (lowest). The 16 amino acids with total contents of 5350.18 μg/g, and two cyclopeptides with total contents of 328.40 μg/g in PR from Jurong, Jiangsu were higher than that from other habitats, according to significant difference test. The contents of all 30 compounds in PR dealt with different processing methods ranged from 3096.58 μg/g to 4146.63 μg/g, in the order: (highest) oven drying 60 °C > oven drying 50 °C > sun drying > sun drying-twisting > oven drying 40 °C (lowest). For different processing methods, the results of total contents of each type of compounds showed that the levels of the two cyclopeptides (272.00 μg/g), 16 amino acids (2960.37 μg/g), and 12 nucleosides (914.27 μg/g) in PR oven dried at 60 °C were significantly higher than that handled by other processing methods, which were marked with different letters. The results also demonstrated that UFLC-MS/MS was a very powerful technique for the quantitative analysis of multicomponent of herbal medicines in terms of time savings, sensitivity, and accuracy.

### 2.6. GRA of the Samples

To further evaluate the variation of cyclopeptides, amino acids, and nucleosides in the all tested samples, GRA was performed according to the contents of 30 bioactive components. The normalization treatment of raw data, dimension of the differences of comparing sequences and reference sequences, correlation coefficient of the evaluated samples and the main components, and correlation degree and weight value of the evaluation samples are given in Table S1. The grey comprehensive evaluation values (ri’) and quality-rankings are listed in Table 4. S1–S5 were collected at different harvesting times, and the quality ranking in PR from different harvesting times is S4 > S1 > S2 > S5 > S3, which indicated that the quality of PR harvested on 6 August 2013 was the better than that of PR from other harvesting times. S6–S10 were collected from five different habitats, and the quality ranking of PR from different habitats is S6 > S8 > S10 > S7 > S9, and PR cultivated in Jurong, Jiangsu showed the best quality compared to PR from other habitats.

S11–S15 were treated with five different processing methods, and the quality ranking of PR handled with different processing methods is S15 > S14 > S12 > S11 > S13, which showed that the quality of PR oven dried at 60 °C was better than that of PR handled by other processing methods. The present method is suitable for the routine analysis and can contribute to quality control of PR from different harvesting times, different habitats, and dealt with using different processing methods.

## 3. Materials and Methods

### 3.1. Chemicals and Reagents

**Figure 2:**
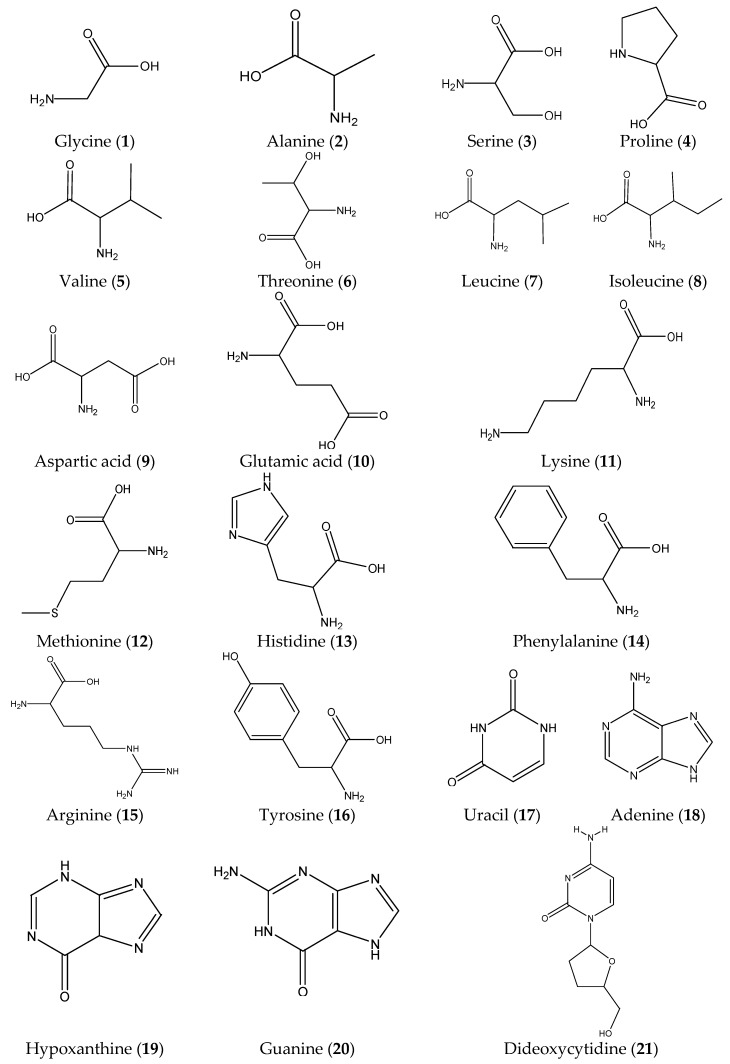

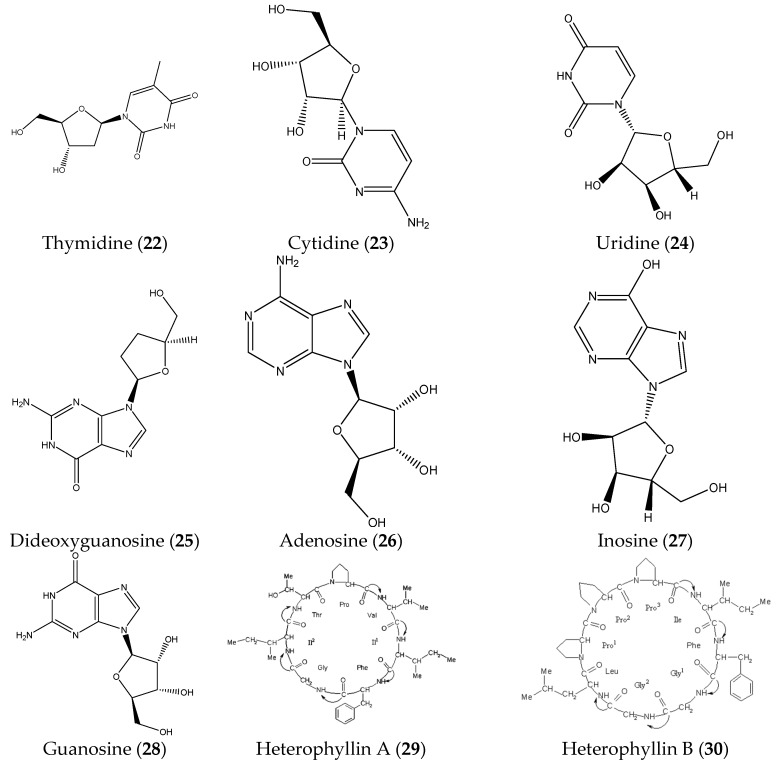
Chemical structures of 30 reference substances.

The reference compounds of glycine (**1**), alanine (**2**), serine (**3**), proline (**4**), valine (**5**), threonine (**6**), leucine (**7**), isoleucine (**8**), aspartic acid (**9**), glutamic acid (**10**), lysine (**11**), methionine (**12**), histidine (**13**), phenylalanine (**14**), arginine (**15**), tyrosine (**16**), uracil (**17**), adenine (**18**), hypoxanthine (**19**), guanine (**20**), dideoxycytidine (**21**), thymidine (**22**), cytidine (**23**), uridine (**24**), dideoxyguanosine (**25**), adenosine (**26**), inosine (**27**), and guanosine (**28**) were purchased from Shanghai Yuanye-Biotechnology Co., Ltd. (Shanghai, China). Heterophyllin A (**29**) and heterophyllin B (**30**) were kindly provided by Professor Ninghua Tan (Kunming Institute of Botany, Chinese Academy of Science, Kunming, China). The structures of the 30 reference compounds are shown in Figure 2. The purity of all compounds by HPLC analysis was greater than 98%. Formic acid of MS grade and acetonitrile were purchased from Merck (Darmstajt, Germany). Ultrapure water was prepared using a Milli-Q water purification system (Millipore, Bedford, MA, USA).

### 3.2. Plant Materials

Fifteen samples from different harvesting times, different habitats, and dealt with using different processing methods were studied in this research. The samples were authenticated by Prof. Xunhong Liu of the Nanjing University of Chinese Medicine. Five samples (S1–S5) were collected at different harvesting times in June to September 2013 (15 June, 9 July, 15 July, 6 August, 12 September) respectively. 

Five samples (S6–S10) were collected from five different habitats (Jurong City, Jiangsu Province, 119°16′49″ N, 31°38′47″ E; Zherong 1 City, Fujian Province, 119°54′2″ N, 27°13′48″ E; Zherong City, Fujian Province, 119°54′2″ N, 27°13′48″ E; Shibing City, Guizhou Province, 108°7′12″ N, 27°1′48″ E; Xuancheng City, Anhui Province, 118°45′ N, 30°56′59″ E) respectively; five samples (S11–S15) were dealt with using different processing methods (sun drying, sun drying-twisting, oven drying 40 °C, oven drying 50 °C, and oven drying 60 °C), respectively. Detailed information is shown in Table 5.

### 3.3. Preparation of Standard Solution

A standard stock solution containing 30 reference standards was prepared in water and their concentrations were as follows: **1**, 2004 ng/mL; **2**, 18684 ng/mL; **3**, 4930 ng/mL; **4**, 6012 ng/mL; **5**, 2012 ng/mL; **6**, 4064 ng/mL; **7**, 988 ng/mL; **8**, 982 ng/mL; **9**, 5940 ng/mL; **10**, 4056 ng/mL; **11**, 4940 ng/mL; **12**, 99.6 ng/mL; **13**, 1976 ng/mL; **14**, 994 ng/mL; **15**, 7952 ng/mL; **16**, 2004 ng/mL; **17**, 3936 ng/mL; **18**, 196 ng/mL; **19**, 1984 ng/mL; **20**, 1476 ng/mL; **21**, 497 ng/mL; **22**, 200 ng/mL; **23**, 3030 ng/mL; **24**, 3048 ng/mL; **25**, 990 ng/mL; **26**, 1972 ng/mL; **27**, 1016 ng/mL; **28**, 2940 ng/mL; **29**, 750 ng/mL; **30**, 5400 ng/mL. This standard stock solution was then diluted with water to a series of appropriate concentrations to generate the calibration curves. The solutions were stored at 4 °C for a day prior to injection.

### 3.4. Preparation of Sample Solutions

The dried roots were pulverized into homogeneous powders (80 mesh). Powder samples (0.1 g) were accurately weighed out and transferred to a 25 mL conical flask equipped with a stopper, and then water (20 mL) was added. After accurately weighing the sample and flask, ultrasonication (250 W, 50 KHz) was performed at 50 °C for 60 min and then water was added to compensate for the weight lost during extraction. After centrifugation (12,000 rpm, 10 min) and filtering (0.22 um membrane filter), the supernatants were stored in a sample plate at 4 °C before injection into the UFLC system for analysis.

### 3.5. Chromatographic and Mass Spectrometric Conditions

Chromatographic analysis was performed on a Shimadzu SIL-20A XR system (Shimadzu, Kyoto, Japan), consisting of a binary solvent delivery system and an autosampler. Separation was performed on a Waters XBridge Amide (2.1 mm × 100 mm, 3.5 μm) column. The mobile phase was composed of water with 0.2% formic acid (A) and acetonitrile with 0.2% formic acid (B) using a gradient elution of 15% A at 0–2.5 min, 15%–50% A at 2.5–5 min, 50% A at 5–7 min, 50%–15% A at 7–8 min, 15% A at 8–11 min. The flow rate was 0.6 mL/min, and the column temperature was set at 30 °C.

Mass spectrometry detection was performed using an API5500 triple quadrupole mass (AB SCIEX, Framingham, MA, USA) equipped with an electrospray ionization (ESI) source operating in the negative ion mode. The ESI-MS spectra were acquired in the multiple reaction monitoring (MRM). The parameters in the source were set as follows: GS1 flow 55 L/min, GS2 flow 55 L/min, CUR flow 40 L/min; gas temperature 550 °C; pressure of nebulizer of MS −4500 V. All MS data were acquired using the Analyst 1.6.2 software to ensure mass accuracy and reproducibility.

### 3.6. Validation of the Method

For the calibration curves, the linearity was verified by plotting the peak areas versus the corresponding concentrations of each analyte. The lowest concentration of working solution for calibration use was diluted with water to a series of appropriate concentrations. The limit of detection (LOD) and limit of quantification (LDQ) of 30 analytes were measured at signal-to-noise values (S/N) of 3 and 10, respectively. The precision of the developed method was determined by the intra- and inter-day variations. For intra-day test, the mixed standard solutions were analyzed for six replicates with a day, while for inter-day test, the solutions were examined for three consecutive days. The relative standard deviation (RSD) was taken as a measure of precision. To confirm the repeatability, six different analytical sample solutions prepared from the same sample (sample 1) were analyzed and variations were expressed by RSD. To evaluate the stability of the solution, one of the sample solution mentioned above was stored at room temperature and analyzed at 0, 2, 4, 8, 12, and 24 h, respectively. A recovery test was utilized to evaluate the accuracy of this method. A known amount of the 30 standards with low (80%), medium (100%), and high (120%) levels were added into a certain amount of samples (0.1 g), and then extracted and analyzed with the same procedures. Three replicate extractives at each level were used to calculate the extraction recovery rates for evaluating the method accuracy. The average recovery percentage was calculated by the formula (1):

Recovery (%) = (total amount after spiking − original amount in sample)/spiked amount × 100%
(1)

### 3.7. Statistical Analysis

GRA was carried out to evaluate quality of PR from different harvesting times, different habitats, and dealt with different processing methods, according to the contents of 30 constituents. The data were also statistically evaluated by one-way ANOVA analysis with the aid of SPSS 19.0 software (SPSS Inc., Chicago, IL, USA) to find the significant differences in the contents of different type of 30 compounds. GRA was carried out by the following steps:

#### 3.7.1. Normalization Treatment of Raw Data

GRA was carried out to evaluate quality of PR from different harvesting times, different habitats, and dealt with different processing methods according to the contents of 30 components. Assume that there were n samples and each sample had m indexes, the X_0_ was the desired sequence (reference sequence) and the evaluation unit sequence was X_i_. The mean numerical calculation method was used to normalize the raw data:

(2) $$x_{i^{\prime }}^{\prime }\left(k\right)=x_{i}\left(k\right)/\frac{1}{m}\overset{m}{\underset{j=1}{\sum }}x_{j}\left(k\right)$$

(i = 0, 1, 2, 3, …, n; k = 0, 1, 2, 3, …, m; n = 25, m = 30 in this experiment).

#### 3.7.2. Calculation of the Correlation Coefficient

The correlation coefficient reflects the accordance between desired sequence and the evaluation unit sequence, the value of correlation coefficient is greater which means that the evaluation unit sequence is closer to desired sequence. The calculation formula of correlation coefficient is:

$$\xi_{i}\left(k\right)=\frac{\underset{i}{\min}\;\underset{k}{\min}\left|X_{0}\left(k\right)-X_{i}\left(k\right)\right|+\rho \;\underset{i}{\max}\;\underset{k}{\max}\left|X_{0}\left(k\right)-X_{i}\left(k\right)\right|}{\left|X_{0}\left(k\right)-X_{i}\left(k\right)\right|+\rho \;\underset{i}{\max}\;\underset{k}{\max}\left|X_{0}\left(k\right)-X_{i}\left(k\right)\right|}$$

ρ = 0.5, $\rho \;\underset{i}{\max}\;\underset{k}{\max}\left|X_{0}\left(k\right)-X_{i}\left(k\right)\right|$ was the secondary minimum differential value and $\underset{i}{\min}\;\underset{k}{\min}\left|X_{0}\left(k\right)-X_{i}\left(k\right)\right|$ was the secondary maximum differential value.

#### 3.7.3. Calculation of the Correlation Degree and Weight Value

It is not convenient to compare the data because there are too much correlation coefficients and the information is dispersive, therefore, it is necessary to concentrate the correlation coefficient to a value and the calculation of mean value is the method of information processing, so that the mean value is the correlation degree. According to the correlation degree ($r_{i}$), the weight value of evaluated samples can be normalized. The formula of correlation degree is:

$$r_{i}=\frac{1}{N}\overset{N}{\underset{k=1}{\sum }}\xi_{i}\left(k\right)$$

#### 3.7.4. The Grey Comprehensive Evaluation Value

$$r_{i}^{\prime }=\frac{1}{N}\overset{N}{\underset{k=1}{\sum }}{\omega К\xi }_{i}\left(k\right)$$

## 4. Conclusions

In this study, an efficient and accurate method was established for the simultaneous quantification of 30 components in PR by using the UFLC-QTRAP-MS/MS technique, which was successfully applied to analyze fifteen PR samples from different harvesting times, different habitats, and dealt with using different processing methods. Furthermore, GRA was performed to evaluate the quality of PR samples according to the contents of 30 marker compounds. The results showed that the quality of the different PR samples was obviously different. The quality of PR harvested on 6 August 2013 was the better than PR from other harvesting times. PR cultivated in Jurong, Jiangsu showed the best quality compared to PR from other habitats. The quality of PR dealt with by oven drying 60 °C was better than PR handled using other processing methods. The proposed method was useful for the overall assessment on the quality of PR, and this study will provide valuable information for revealing the dynamic change law of metabolite accumulation in PR, choosing the suitable harvesting time and reasonable processing method of PR, and exploring the mechanisms responsible for its quality.

## Figures and Tables

**Table 1:** Retention time, related MS data of the target compounds.

Coumpounds	RT (min)	[M + H]^+^ (*m*/*z*)	Precursor Ion	Product Ion	FV	CE
Heterophyllin A	0.50	728.43	728.4	70.05	211	119
Heterophyllin B	0.55	779.44	779.39	70.05	226	117
Uracil	0.75	112.09	113.04	70	111	21
Thymidine	0.78	242.23	243.1	127.07	61	13
Hypoxanthine	1.11	136.11	137.05	137.05	51	24
Uridine	1.13	244.2	244.9	113	103	13
Adenine	1.31	135.13	136.06	136	51	24
Adenosine	1.39	267.24	268.1	136.07	86	23
Phenylalanine	1.4	165.19	166.1	120.05	56	14
Inosine	1.59	268.23	269	137.07	46	15
Valine	1.66	117.15	118.09	72.06	54	10
Methionine	1.72	149.21	150.06	104.03	91	10
Dideoxyguanosine	1.79	267.2	268.1	152.1	61	15
Leucine	1.81	131.18	132.1	86.05	98	10
Tyrosine	1.85	182.1	182.16	136.08	46	17
Dideoxycytidine	1.87	227.3	228.2	112.05	76	13
Isoleucine	1.92	131.18	132.1	86.05	64	10
Guanine	1.95	151.12	152	135	51	25
Proline	2.31	115.13	116.07	70.02	68	10
Guanosine	2.45	283.24	284.3	152	62	15
Alanine	2.66	89.09	90.06	44.02	79	10
Cytidine	2.93	243.22	244.09	112	61	10
Glycine	3.45	75.07	76.04	30	73	6
Threonine	3.98	119.12	120.07	74	93	20
Aspartic acid	4.25	133.1	134.05	87.96	59	10
Serine	4.42	105.09	106.05	59.99	67	8
Arginine	5.07	174.2	175.12	70.02	88	18
Histidine	5.11	155	156.08	110.03	95	16
Lysine	5.12	146.19	147.11	83.91	66	14
Glutamic acid	5.14	147.13	147.08	83.92	83	14

**Table 2:** Regression equation, LOD and LOQ, precision, repeatability, stability and recovery of 30 investigated compounds.

No.	Compounds	Regression Equation	*r*^2^	Liner Range (ng/mL)	LOQ (ng/mL)	LOD (ng/mL)	Precision RSD (%)		Repeatability RSD (%) (*n* = 6)	Stability RSD (%)	Recovery (%) (*n* = 3)					
							Intar-Day (*n* = 6)	Inter-Day (*n* = 6)			Low		Medium		High	
											Mean	RSD	Mean	RSD	Mean	RSD
1	Glycine	Y = 138X + 4880	0.9991	20.04–2004	10.02	4.01	2.09	3.47	2.66	3.70	98.75	1.4	99.7	1.81	100.79	1.19
2	Alanine	Y = 1860X + 222000	0.9999	186.84–18684	93.40	31.13	2.49	2.59	1.45	1.02	100.78	0.92	102	1.11	101.32	1.2
3	Serine	Y = 872X + 52100	0.9996	4.93–4930	4.93	1.64	2.10	3.48	3.31	2.67	97.89	1.72	102.76	2.55	101.3	1.52
4	Proline	Y = 10100X − 459000	0.9995	30.06–6012	15.03	7.52	1.82	2.54	1.38	3.67	103.53	1.06	102.22	2.72	99.34	2.3
5	Valine	Y = 10100X + 136000	0.9992	10.06–2012	0.40	0.13	2.96	3.07	2.12	3.20	99.05	2.24	101.64	2.35	102.28	1.84
6	Threonine	Y = 1250X + 70400	0.9991	81.28–4064	40.64	20.32	2.01	2.61	3.14	1.33	101.68	1.79	101.08	1.76	102.58	2.39
7	Leucine	Y = 27700X + 403000	0.9979	4.94–988	1.98	0.66	2.1	3.79	2.89	3.77	103.04	1.09	102.19	2.11	99.17	2.75
8	Isoleucine	Y = 27900X + 403000	0.9979	4.91–982	1.96	0.65	2.88	4.07	1.14	1.11	104.36	2.31	100.87	4.72	100.44	4.18
9	Aspartic acid	Y = 1120X + 36200	0.9998	29.7–5940	11.88	3.57	1.55	1.93	1.83	3.57	98.92	2.62	98.97	2.34	99.89	1.71
10	Glutamic acid	Y = 4740X + 99900	1.0000	20.28–4056	4.06	0.81	1.02	2.57	2.46	3.12	101.76	2.39	100.2	1.73	99.86	1.58
11	Lysine	Y = 3040X + 43100	0.9996	24.70–4940	4.94	2.47	0.61	2.98	2.53	1.34	98.74	4.23	101.59	2.56	99.41	1.79
12	Methionine	Y = 4160X + 3600	0.9998	0.996–99.6	0.50	0.20	1.75	3.37	1.01	3.84	98.09	4.76	102.97	4.52	99.74	3.04
13	Histidine	Y = 12400X + 91600	1.0000	19.76–1976	9.88	3.95	1.55	2.43	2.55	3.58	97.8	2.31	100.08	1.52	100.83	1.47
14	Phenylalanine	Y = 18800X + 102000	0.9999	4.97–994	1.99	0.50	1.31	3.58	1.47	2.40	99.03	3.06	101.54	3.63	100.81	4.04
15	Arginine	Y = 5690X + 430000	0.9998	19.88–7952	1.08	0.32	0.71	1.95	2.36	2.24	99.27	1.51	99.84	1.61	99.03	1.25
16	Tyrosine	Y = 9780X + 179000	0.9998	20.04–2004	2.00	0.40	2.16	3.16	1.62	2.70	99.29	2.44	99.55	2.39	101.53	2.17
17	Uracil	Y = 299X + 35600	0.9990	1.97–3936	0.79	0.32	3.3	4.39	2.89	2.11	104.85	2.67	99.53	3.43	97.94	3.18
18	Adenine	Y = 5810X + 461000	0.9947	1.96–196	1.96	0.98	1.03	2.49	3.93	3.87	104.3	1.66	101.64	4.63	98.17	2.67
19	Hypoxanthine	Y = 2470X + 59300	0.9993	19.84–1984	9.92	3.97	3.34	4.08	3.71	1.71	99.67	1.59	99.93	1.02	99.99	1.05
20	Guanine	Y = 3780X + 98000	0.9992	14.76–1476	5.90	2.36	1.05	3.17	3.65	3.71	103.61	1.61	101.93	2.16	101.35	2.14
21	Dideoxycytidine	Y = 19900X + 46500	0.9999	4.97–497	1.99	0.50	2.01	3.44	2.81	2.12	98.96	2.77	101.58	4.83	98.45	2.01
22	Thymidine	Y = 2940X + 3200	0.9994	2.00–200	1.00	0.50	1.92	3.12	3.13	3.34	100.21	3.99	103.16	3.58	95.98	1.72
23	Cytidine	Y = 17600X + 361000	0.9999	15.15–3030	1.52	0.61	2.98	3.61	3.44	2.43	100.04	3.26	99.82	2.82	99.49	2.04
24	Uridine	Y = 1260X + 28000	0.9992	30.48–3048	15.24	3.05	2.18	3.32	3.34	3.99	97.69	2.53	100.95	2.14	99.81	2.95
25	Dideoxyguanosine	Y = 13000X + 143000	0.9997	4.95–990	0.99	0.25	1.94	4.28	3.52	2.67	103.88	2.35	103.01	1.51	101.45	4.16
26	Adenosine	Y = 25400X + 944000	0.9994	19.72–1972	0.99	0.39	2.54	3.39	2.53	2.66	100.91	1.36	99.72	1.08	99.76	1.17
27	Inosine	Y = 16900X + 127000	0.9994	10.16–1016	5.08	2.03	2.14	2.96	2.09	3.69	101.97	2.02	102.79	4.81	95.68	2.02
28	guanosine	Y = 15600X + 346000	0.9999	14.70–2940	0.74	0.29	3.25	4.18	2.86	1.20	100.14	2.05	99.25	1.49	100.56	1.66
29	Heterophyllin A	Y = 4190X + 10700	0.9996	1.25–750	0.75	0.38	2.1	3.35	2.67	3.28	101.52	3.32	97.57	3.34	99.96	1.96
30	Heterophyllin B	Y = 3480X − 40800	0.9996	5.4–5400	0.54	0.22	3.28	4.37	2.55	2.87	100.42	3.6	101.35	1.99	102.45	1.1

**Table 3:** Contents of 30 compounds in PR (μg/g, mean ± SD, *n* = 3).

Analyte	Different Harvesting Times					Different Habitats					Different Processing Methods				
	S1 ^a^	S2	S3	S4	S5	S6	S7	S8	S9	S10	S11	S12	S13	S14	S15
1 ^b^	104.09 ± 5.85	77.34 ± 4.21	39.23 ± 9.39	117.41 ± 7.27	64.98 ± 1.63	114.33 ± 3.56	76.467 ± 4.05	67.99 ± 19.17	42.87 ± 2.64	100.42 ± 6.25	36.00 ± 3.41	41.267 ± 2.19	33.73 ± 5.66	45.53 ± 1.33	56.20 ± 1.71
2	1028.67 ± 23.35	910.67 ± 12.86	824.00 ± 20.88	2468.06 ± 81.89	1296.67 ± 27.15	1872.67 ± 19.73	978.00 ± 44.14	1250.67 ± 27.30	1130.00 ± 6.93	1077.33 ± 22.30	568.00 ± 7.21	531.33 ± 36.02	237.33 ± 3.06	696.00 ± 5.29	634.00 ± 10.58
3	118.40 ± 3.42	107.80 ± 11.72	129.53 ± 15.60	281.33 ± 11.72	131.00 ± 14.91	211.13 ± 28.46	139.67 ± 7.18	143.53 ± 6.45	87.93 ± 12.07	106.93 ± 13.06	52.60 ± 1.31	40.53 ± 6.35	6.10 ± 4.09	27.33 ± 2.66	21.20 ± 1.59
4	157.67 ± 5.83	148.60 ± 3.70	109.00 ± 0.87	9.29 ± 0.042	300.67 ± 6.43	443.33 ± 4.16	136.53 ± 3.45	195.13 ± 5.16	114.33 ± 0.76	240.67 ± 3.06	106.87 ± 2.32	80.73 ± 7.92	21.67 ± 0.46	106.47 ± 1.33	218.00 ± 4.00
5	118.93 ± 1.51	139.53 ± 2.80	84.13 ± 1.81	156.55 ± 1.60	74.73 ± 0.64	114.067 ± 2.37	78.2 ± 1.56	85.07 ± 2.00	75.00 ± 1.64	101.00 ± 2.31	71.00 ± 1.60	75.80 ± 3.94	41.67 ± 0.95	70.27 ± 2.08	74.80 ± 3.29
6	107.33 ± 2.91	141.13 ± 4.23	124.27 ± 3.06	250.00 ± 2.71	134.60 ± 1.91	206.67 ± 2.31	98.60 ± 7.32	127.00 ± 2.27	132.33 ± 5.25	126.47 ± 3.14	122.67 ± 2.00	125.13 ± 2.27	111.87 ± 2.20	146.60 ± 2.09	169.20 ± 2.31
7	89.93 ± 0.64	80.87 ± 1.01	39.00 ± 0.20	108.4 ± 1.91	53.13 ± 0.95	83.73 ± 2.20	65.93 ± 1.17	65.73 ± 0.76	53.20 ± 1.04	56.33 ± 0.83	27.67 ± 1.14	31.27 ± 3.72	9.48 ± 0.087	24.067 ± 0.12	31.80 ± 1.91
8	89.40 ± 0.53	80.40 ± 0.92	38.80 ± 0.20	107.67 ± 1.81	52.73 ± 0.95	83.27 ± 2.14	65.53 ± 1.17	65.33 ± 0.76	52.87 ± 0.99	56.00 ± 0.92	27.47 ± 1.14	31.07 ± 3.72	9.39 ± 0.075	23.87 ± 0.12	31.53 ± 1.90
9	223.33 ± 13.01	240.67 ± 14.05	210.53 ± 11.25	513.33 ± 19.63	276.00 ± 4.00	395.33 ± 9.02	173.73 ± 5.82	250.67 ± 16.29	160.6 ± 0.60	202.00 ± 2.00	90.80 ± 6.77	80.33 ± 0.42	39.67 ± 2.80	79.40 ± 2.42	58.13 ± 1.81
10	115.07 ± 2.02	116.4 ± 6.51	94.27 ± 4.02	211.2 ± 12.24	98.4 ± 2.46	154.07 ± 2.84	62.67 ± 0.81	104.80 ± 4.73	82.53 ± 4.31	108.20 ± 1.73	54.80 ± 1.78	50.00 ± 2.42	29.93 ± 1.50	53.27 ± 0.76	50.33 ± 0.42
11	123.20 ± 4.04	126.80 ± 4.19	98.80 ± 6.45	224.00 ± 0.00	104.40 ± 3.27	169.20 ± 1.91	67.60 ± 2.31	112.60 ± 2.42	86.07 ± 2.48	121.80 ± 0.69	59.33 ± 3.11	54.40 ± 4.06	33.33 ± 1.67	59.07 ± 1.30	54.80 ± 2.60
12	3.76 ± 0.18	0.61 ± 0.20	1.00 ± 0.080	6.01 ± 1.72	4.79 ± 0.012	8.69 ± 0.69	2.33 ± 0.11	5.41 ± 0.59	0.37 ± 0.071	1.32 ± 0.31	0.27 ± 0.11	0.49 ± 0.00	0.29 ± 0.096	0.30 ± 0.12	0.23 ± 0.030
13	75.13 ± 1.86	108.00 ± 2.27	85.93 ± 2.14	216.67 ± 4.16	124.13 ± 4.22	177.80 ± 2.51	83.80 ± 2.88	295.33 ± 7.02	189.33 ± 4.00	45.73 ± 0.99	107.00 ± 1.25	103.00 ± 1.60	100.53 ± 2.54	130.67 ± 2.87	110.27 ± 1.67
14	33.80 ± 13.56	46.47 ± 0.61	27.07 ± 0.50	33.53 ± 4.11	41.73 ± 0.76	55.67 ± 18.25	39.53 ± 19.15	35.00 ± 2.62	43.87 ± 0.23	27.53 ± 11.68	32.53 ± 0.31	38.27 ± 1.29	22.93 ± 0.70	33.33 ± 0.50	57.00 ± 0.72
15	1352.67 ± 22.03	1230.67 ± 160.81	1288.00 ± 119.31	1205.33 ± 1.15	1422.67 ± 90.56	1140.67 ± 15.28	1172.00 ± 36.39	1196.00 ± 23.07	1179.33 ± 134.23	1304.00 ± 55.46	1257.33 ± 79.10	1285.33 ± 41.05	1366.00 ± 58.41	1357.33 ± 71.06	1319.33 ± 18.04
16	82.37 ± 35.21	55.13 ± 25.84	27.49 ± 0.66	250.17 ± 5.93	67.53 ± 3.19	119.56 ± 59.61	77.45 ± 5.86	101.79 ± 1.82	39.07 ± 3.89	61.25 ± 0.78	45.60 ± 25.46	82.40 ± 4.53	60.87 ± 0.42	68.60 ± 0.58	73.53 ± 0.76
Total	3823.75 ± 37.07 ^c^	3611.08 ± 185.73 ^c^	3221.06 ± 84.97 ^d^	6158.95 ± 139.62 ^a^	4248.17 ± 51.22 ^b^	5350.18 ± 33.29 ^a^	3318.05 ± 11.07 ^d^	4102.06 ± 75.32 ^b^	3469.71 ± 125.79 ^d^	3736.99 ± 40.10 ^c^	2659.93 ± 93.99 ^b^	2651.36 ± 77.58 ^b^	2124.80 ± 43.94 ^c^	2922.10 ± 73.45 ^a^	2960.37 ± 24.80 ^a^
17	66.07 ± 2.95	17.5 ± 4.50	9.16 ± 5.42	11.97 ± 2.43	2.18 ± 2.11	12.98 ± 9.39	8.46 ± 2.51	11.44 ± 2.97	20.12 ± 27.33	42.4 ± 8.46	3.42 ± 0.34	3.74 ± 1.06	2.78 ± 1.30	8.70 ± 1.50	2.58 ± 1.30
18	10.74 ± 1.22	6.76 ± 2.06	9.13 ± 0.60	15.35 ± 0.32	9.25 ± 0.85	15.09 ± 0.53	6.99 ± 0.84	13.60 ± 0.97	3.39 ± 0.55	10.31 ± 0.66	10.90 ± 0.53	4.90 ± 2.53	3.92 ± 1.98	6.63 ± 1.12	7.03 ± 2.51
19	213.33 ± 4.62	20.95 ± 7.56	10.64 ± 0.60	26.00 ± 0.69	17.36 ± 1.56	31.93 ± 15.37	13.79 ± 2.61	38.29 ± 16.70	15.59 ± 6.51	24.87 ± 2.83	22.87 ± 1.03	14.75 ± 6.28	16.27 ± 6.76	13.09 ± 8.25	32.93 ± 0.58
20	49.60 ± 1.59	13.29 ± 0.076	11.21 ± 0.49	50.40 ± 1.56	16.61 ± 1.02	33.40 ± 0.87	17.25 ± 0.56	30.47 ± 0.46	3.09 ± 0.00	22.13 ± 0.12	10.95 ± 0.74	11.59 ± 0.89	15.69 ± 1.15	16.61 ± 0.67	17.23 ± 0.63
21	3.02 ± 0.072	7.39 ± 0.095	6.97 ± 0.46	21.92 ± 0.23	7.50 ± 0.17	15.48 ± 0.060	7.61 ± 0.50	9.31 ± 0.067	1.56 ± 0.071	7.85 ± 0.77	7.47 ± 0.17	8.62 ± 0.63	7.99 ± 0.19	7.18 ± 0.21	6.77 ± 0.11
22	37.40 ± 10.86	15.54 ± 0.52	14.23 ± 13.89	41.80 ± 13.34	15.08 ± 2.48	30.33 ± 5.20	20.15 ± 1.37	23.47 ± 8.68	6.14 ± 7.43	20.80 ± 2.31	16.25 ± 1.40	19.67 ± 0.85	19.63 ± 1.61	16.64 ± 0.98	14.84 ± 0.26
23	64.27 ± 2.00	68.27 ± 1.75	57.18 ± 1.70	193.90 ± 1.10	68.20 ± 74.01	134.39 ± 1.57	130.13 ± 4.32	200.73 ± 4.61	60.60 ± 0.72	112.40 ± 1.51	148.27 ± 3.01	128.07 ± 3.92	123.60 ± 1.04	139.20 ± 2.84	156.33 ± 2.83
24	262.00 ± 6.93	160.73 ± 8.27	98.47 ± 0.99	234.00 ± 3.46	104.07 ± 3.35	174.87 ± 4.24	162.93 ± 10.00	218.67 ± 4.62	155.93 ± 6.33	155.07 ± 5.52	176.87 ± 2.42	167.20 ± 5.33	163.00 ± 7.41	176.20 ± 2.11	193.07 ± 4.00
25	31.20 ± 1.06	15.71 ± 0.47	14.13 ± 0.58	44.33 ± 1.10	12.53 ± 0.12	26.67 ± 0.61	18.11 ± 0.75	23.13 ± 0.31	3.34 ± 0.035	15.94 ± 0.20	12.85 ± 0.14	16.10 ± 0.53	16.01 ± 0.33	14.67 ± 0.10	13.57 ± 0.24
26	141.87 ± 2.19	162.47 ± 2.20	108.00 ± 5.64	259.33 ± 7.57	108.13 ± 2.72	163.60 ± 1.44	182.47 ± 3.97	241.33 ± 3.06	146.40 ± 5.05	157.53 ± 2.34	168.33 ± 4.10	171.53 ± 7.17	159.87 ± 2.60	166.20 ± 7.22	176.20 ± 3.47
27	34.27 ± 0.61	10.71 ± 0.25	6.55 ± 0.012	19.01 ± 0.32	6.38 ± 0.19	10.67 ± 0.076	12.42 ± 0.49	17.77 ± 0.095	9.55 ± 0.042	10.29 ± 0.061	11.31 ± 0.44	11.50 ± 0.41	10.65 ± 0.33	10.97 ± 0.14	11.71 ± 0.34
28	228.00 ± 9.17	199.47 ± 4.24	136.87 ± 4.71	366.67 ± 13.32	139.73 ± 1.79	252.67 ± 1.15	253.33 ± 12.22	349.33 ± 11.02	130.80 ± 0.60	193.87 ± 2.12	282.00 ± 3.46	247.33 ± 7.02	236.00 ± 2.00	251.33 ± 5.03	282.00 ± 2.00
Total	1141.76 ± 11.82 ^b^	698.87 ± 22.87 ^c^	482.54 ± 14.14 ^d^	1284.68 ± 22.44 ^a^	507.02 ± 2.10 ^d^	902.07 ± 13.08 ^b^	833.63 ± 30.40 ^c^	1177.45 ± 19.17 ^a^	556.51 ± 29.15 ^d^	773.46 ± 15.4 ^c^	871.48 ± 9.64 ^b^	805.00 ± 25.59 ^c,d^	775.40 ± 15.88 ^d^	827.41 ± 5.77 ^c^	914.27 ± 4.98 ^a^
29	19.20 ± 0.71	20.10 ± 0.97	13.43 ± 0.50	26.47 ± 0.58	12.21 ± 0.29	19.07 ± 0.70	104.07 ± 13.40	59.20 ± 3.30	26.60 ± 0.80	56.00 ± 1.78	20.87 ± 0.81	18.48 ± 1.08	18.38 ± 0.92	22.40 ± 1.22	24.67 ± 1.01
30	274.00 ± 5.29	296.00 ± 15.62	234.00 ± 6.93	388.00 ± 10.58	220.00 ± 0.00	309.33 ± 10.26	11.55 ± 0.84	4.98 ± 0.11	217.33 ± 5.77	102.67 ± 7.56	198.73 ± 8.25	174.07 ± 10.02	178.00 ± 1.74	220.00 ± 2.00	247.33 ± 3.06
Total	293.20 ± 5.92 ^b^	316.10 ± 16.58 ^b^	247.43 ± 6.53 ^c^	414.47 ± 10.41 ^a^	232.21 ± 0.29 ^c^	328.40 ± 9.81 ^a^	115.61 ± 14.15 ^d^	64.18 ± 3.29 ^e^	243.93 ± 5.83 ^b^	158.67 ± 7.14 ^c^	219.60 ± 8.99 ^c^	192.55 ± 10.88 ^d^	196.38 ± 0.90 ^d^	242.40 ± 1.04 ^b^	272.00 ± 4.06 ^a^

^a^ The sample No. is same as in Table 5; ^b^ The analyte No. is the same as in Table 2; ^c^ Values followed by the same letter in the same row are not significantly different (*p* < 0.05).

**Table 4:** Quality sequencing of the samples.

Sample	Grey Comprehensive Evaluation Value *(r_i_’)*	Quality-Ranking
S1	0.0260	2
S2	0.0231	3
S3	0.0211	5
S4	0.0311	1
S5	0.0227	4
S6	0.0300	1
S7	0.0222	4
S8	0.0262	2
S9	0.0201	5
S10	0.0228	3
S11	0.0276	4
S12	0.0278	3
S13	0.0237	5
S14	0.0281	2
S15	0.0298	1

**Table 5:** Summary of information of samples. S1–S5 were collected at different harvesting times. S6–S10 were collected from five different habitats. S11–S15 were dealt with by five different processing methods.

Sample No.	Habitats	Harvesting Time	Processing Method
S1	Jurong, Jiangsu	15 June 2013	sun drying
S2	Jurong, Jiangsu	9 July 2013	sun drying
S3	Jurong, Jiangsu	15 July 2013	sun drying
S4	Jurong, Jiangsu	6 August 2013	sun drying
S5	Jurong, Jiangsu	12 September 2013	sun drying
S6	Jurong, Jiangsu	10 August 2013	sun drying
S7	Zherong 1, Fujian	10 August 2013	sun drying
S8	Zherong 2, Fujian	10 August 2013	sun drying
S9	Shibing, Guizhou	10 August 2013	sun drying
S10	Xuancheng, Anhui	10 August 2013	sun drying
S11	Jurong, Jiangsu	10 August 2013	sun drying
S12	Jurong, Jiangsu	10 August 2013	sun drying-twisting
S13	Jurong, Jiangsu	10 August 2013	oven drying 40 °C
S14	Jurong, Jiangsu	10 August 2013	oven drying 50 °C
S15	Jurong, Jiangsu	10 August 2013	oven drying 60 °C

## Supplementary Materials

Supplementary materials can be accessed at: http://www.mdpi.com/1420-3049/22/1/13/s1.
